# Genome-Wide Analysis of Long Noncoding RNA (lncRNA) Expression in Hepatoblastoma Tissues

**DOI:** 10.1371/journal.pone.0085599

**Published:** 2014-01-17

**Authors:** Rui Dong, Deshui Jia, Ping Xue, Ximao Cui, Kai Li, Shan Zheng, Xianghuo He, Kuiran Dong

**Affiliations:** 1 Department of Pediatric Surgery, Children's Hospital of Fudan University and The Key Laboratory of Neonatal Disease, Chinese Ministry of Health, Shanghai, China; 2 State Key Laboratory of Oncogenes and Related Genes, Shanghai Cancer Institute, Ren Ji Hospital, Shanghai Jiao Tong University School of Medicine, Shanghai, China; Harbin Institute of Technology, China

## Abstract

Long noncoding RNAs (lncRNAs) have crucial roles in cancer biology. We performed a genome-wide analysis of lncRNA expression in hepatoblastoma tissues to identify novel targets for further study of hepatoblastoma. Hepatoblastoma and normal liver tissue samples were obtained from hepatoblastoma patients. The genome-wide analysis of lncRNA expression in these tissues was performed using a 4×180 K lncRNA microarray and Sureprint G3 Human lncRNA Chips. Quantitative RT-PCR (qRT-PCR) was performed to confirm these results. The differential expressions of lncRNAs and mRNAs were identified through fold-change filtering. Gene Ontology (GO) and pathway analyses were performed using the standard enrichment computation method. Associations between lncRNAs and adjacent protein-coding genes were determined through complex transcriptional loci analysis. We found that 2736 lncRNAs were differentially expressed in hepatoblastoma tissues. Among these, 1757 lncRNAs were upregulated more than two-fold relative to normal tissues and 979 lncRNAs were downregulated. Moreover, in hepatoblastoma there were 420 matched lncRNA-mRNA pairs for 120 differentially expressed lncRNAs, and 167 differentially expressed mRNAs. The co-expression network analysis predicted 252 network nodes and 420 connections between 120 lncRNAs and 132 coding genes. Within this co-expression network, 369 pairs were positive, and 51 pairs were negative. Lastly, qRT-PCR data verified six upregulated and downregulated lncRNAs in hepatoblastoma, plus endothelial cell-specific molecule 1 (ESM1) mRNA. Our results demonstrated that expression of these aberrant lncRNAs could respond to hepatoblastoma development. Further study of these lncRNAs could provide useful insight into hepatoblastoma biology.

## Introduction

Hepatoblastoma is an uncommon liver malignancy in infants and children, accounting for just over 1% of pediatric cancers [1], but with increasing incidence in North America and Europe [2]. The disease is most commonly diagnosed during a child's first three years of life [3], while only 5% of new hepatoblastoma cases are found in children older than 4 years. The disease occurs significantly more frequently in boys than in girls, for reasons that remain to be determined [4].

Hepatoblastoma originates from immature liver precursor cells [2]. Histologically, hepatoblastoma can be divided into epithelial or mixed epithelial/mesenchymal tissues. The majority of hepatoblastoma is epithelial in origin and consists of a mixture of embryonal and fetal cell types. Approximately 5% of hepatoblastoma is of the small cell undifferentiated subtype and is associated with a poor prognosis [5]. To date, surgical resection, adjuvant chemotherapy, and liver transplantation are the only options to treat hepatoblastoma. Thus, we urgently need novel strategies to improve our understanding of the biology of hepatoblastoma, and to provide targets for therapy or early detection of the disease.

A study of Children's Cancer Group [6] showed a significant linkage between hepatoblastoma and maternal and paternal exposure to metals. Other studies associated hepatoblastoma with Beckwith-Weidemann syndrome, familial adenomatosis polypi, and low birth weight [1], [7]. Genetic syndromes are associated with approximately 15% of hepatoblastoma and the recognition and delineation of these syndromes could help us determine their origin in family members [8].

There is evidence to suggest that lncRNAs, a subset of non-coding RNAs >200 nucleotides in length, participate in transcriptional, epigenetic, or post-transcriptional regulation of gene expression [9]. Altered long non-coding RNA (lncRNA) levels have been observed in gastric cancer [10], colorectal cancer [11], renal cell carcinoma [12], and hepatocellular carcinoma [13], indicating that aberrant expression of certain lncRNAs contributes to carcinogenesis. Many lncRNAs have been found using large-scale analyses of full-length cDNA sequences and other methods, in humans and mice [14]. However, although over a decade's research has led to considerable progress in understanding lncRNAs, the precise function of most remains unknown.

We wondered if alterations in lncRNAs have a role in hepatoblastoma. To identify novel targets for further study of hepatoblastoma, we analyzed hepatoblastoma tissue samples and paired distant noncancerous tissues to profile differentially expressed lncRNAs and mRNAs in the disease.

## Materials and Methods

### Patient samples and RNA extraction

Between March 2009 and March 2010 we prospectively recruited four patients with primary hepatoblastoma from Children's Hospital of Fudan University. Hepatoblastoma and paired distant noncancerous tissue samples were obtained surgically. Four patients underwent partial hepatectomy and tissue pathology confirmed hepatoblastoma with more than 80% viable tumor cells. The patients did not undergo any chemotherapy or other forms of therapy. Clinical data were obtained retrospectively from clinical files (Table 1). The Ethics Committee of Children's Hospital of Fudan University approved our study. The parents of all participants provided written informed consent prior to enrollment.

**Table 1:** Distribution of study subjects and serum AFP levels.

Case	Age (months)*	Gender	Diagnosis Type	AFP(ng/ml)	Viral hepatitis
501	7	Male	Mixed embryonal/fetal subtype	68490	None
502	23	Female	Mixed embryonal/fetal subtype	>121000	None
508	11	Female	Epithelial type	>121000	None
509	10	Female	Epithelial type	>121000	None

*at serum sample day.

Total cellular RNA was isolated from the fresh primary hepatoblastoma and paired distant noncancerous tissues using an RNeasy Mini Kit (Qiagen, Hilden, Germany) in accordance with the manufacturer's protocol, and then quantified using a NanoDrop ND-1000 spectrophotometer (Thermo Fisher Scientific, Waltham, MA). The RNA integrity of each sample was assessed using standard denaturing agarose gel electrophoresis.

### Microarray and data analyses

#### lncRNA and mRNA microarray

Human 4×180 K lncRNA arrays manufactured by Agilent Technologies (Santa Clara, CA) and Sureprint G3 Human lncRNA Chip (i.e., BT1000312) reportedly represented all long transcripts, both protein coding mRNAs and lncRNAs, in the human genome, more than 46 506 lncRNAs and 30 656 mRNAs from NCBI RefSeq, UCSC, RNAdb, lncRNAs reported in the literature, and ultraconserved regions. Each transcript was represented by using 1–5 probes to improve statistical confidence. The lncRNA expression data have been deposited into Gene Expression Omnibus (GEO) under accession number GSE51701.

#### RNA labeling and array hybridization

Total RNA (200 ng each) from these tissue samples was reversely transcribed into cDNA using an RNA Spike In Kit with one-color (Agilent) in the presence of 0.8 µL of Random Primer and 2 µL of Spike Mix. These cDNA samples were then cleaned and labeled in accordance with the Agilent Gene Expression Analysis protocol using Low Input Quick-Amp Labeling Kit, one-color (Agilent). These labeled cDNA samples were used as probes to hybridize to microarrays for 17 h at 65°C using an Agilent Gene Expression Hybridization Kit in hybridization chamber gasket slides (Agilent). After hybridization, the microarrays were washed with an Agilent Wash Buffer kit (Agilent) and scanned with an Agilent microarray scanner.

#### Data analysis

The microarrays were scanned at 5 µm/pixel resolutions using an Agilent microarray scanner piloted by GenePix Pro 6.0 software (Axon). Scanned images (TIFF format) were then imported into Agilent Feature Extraction software for grid alignment and expression data analysis. Expression data were normalized by a quantile normalization and the Robust Multichip Average (RMA) algorithm that was included in the Agilent software. Probe-level files and mRNA-level files were generated after normalization. All gene-level files were imported into Agilent GeneSpring GX software (version 11.5.1) for further analysis. Differentially expressed lncRNAs and mRNAs were identified through fold change filtering.

### Gene function analysis

The predicted target genes above were input into the Database for Annotation, Visualization and Integrated Discovery (DAVID; http://david.abcc.ncifcrf.gov/), which utilized Gene Ontology (GO) to identify the molecular function represented in the gene profile [15]. Furthermore, we also used the KEGG (Kyoto Encyclopedia of Genes and Genomes) database (http://www.genome.ad.jp/kegg/) and BioCarta (http://www.biocarta.com) to analyze the potential functions of these target genes in the pathways [16], [17]. The lower the *P*-value, the more significant the correlation; the recommended *P*-value cut-off is 0.05.

### Analysis of lncRNA-mRNA regulatory network

To associate the lncRNAs with direct regulated expression of target mRNAs, we superimposed lncRNA target predictions onto the lncRNA-mRNA correlation network. The resulting network was defined as an lncRNA-mRNA regulatory network. A direct connection between an lncRNA and an mRNA was represented as a solid line (trans interaction).

### Quantitative RT-PCR

Total cellular RNA was isolated from hepatoblastoma and normal tissues using TRIzol reagent (Invitrogen, Carlsbad, CA, USA) and then reversely transcribed using a PrimeScript RT reagent Kit with gDNA Eraser (Perfect Real Time) (TaKaRa, Dalian, China) in accordance with the manufacturer's instructions. The expressions of selected upregulated lncRNAs (i.e., TCONS-00090092-MEG3, TCONS-l2-00000179, TCONS-l2-00014091, TCONS-l2-00004424, TCONS-l2-00021262, and TCONS-00014978) and under-regulated lncRNAs (TCONS-l2-00018070, TCONS-l2-00018071, TCONS-l2-00006843, TCONS-l2-00030560, TCONS-l2-00020565, and TCONS-00024647), and a pair of lncRNAs and mRNA (TCONS_00014512 and endothelial cell-specific molecule 1 [ESM1]) were analyzed using qRT-PCR with a SYBRGreen PCR kit (TaKaRa). Glyceraldehyde 3-phosphate dehydrogenase (GAPDH) mRNA was used as an internal control. The primers are listed in Table 2. For quantitative results, expression of each lncRNA was represented as a fold change using the 2-ΔΔCt method and then statistically analyzed.

**Table 2:** Primers used for qRT-PCR analysis of lncRNA and mRNA levels.

Target ID	Forward primer	Reverse primer	Product length (bp)	T_m_ (°C)
TCONS_00090092_MEG3	5′-ACACTTGCTGTCTTCCTT-3′	5′-CCAGGTCAGGAACTTTGT-3′	102	60
TCONS_l2_00018070	5′-TCCAACAGATCGACAACG-3′	5′-GTACTTCGATGTCACGGG-3′	159	60
TCONS_l2_00018071	5′-GTGATTCACGAGGTGCAG-3′	5′-CTACCTTAGGGATGCGGA-3′	103	60
TCONS_l2_00006843	5′-TACAGGGTGCCAGATAGATT-3′	5′-AGTCACAGTACTAACTGTCCCA-3′	103	60
TCONS_l2_00000179	5′-GGAGGAAGATGCAGAGTCA-3'	5′-TTTCTGTGGAACCTTGCTAC-3′	106	60
TCONS_l2_00014091	5′-GACTGTATTAACCAAGTCTCCC-3′	5′-CCTATGTTGTAGCCATAGCTG-3′	110	60
TCONS_l2_00004424	5′-AGACGTGAAGATCAAGTCC-3′	5′-TGCTTCTGCACTGGCATA-3′	132	60
TCONS_l2_00021262	5′-AAGTAGGTACTTCTTATGGCAG-3′	5′-TGAGTGAGTCCACAGATTATCC-3'	103	60
TCONS_l2_00030560	5′-CCCAGCACAAGAGGGATA-3′	5′-ATTCCACAGTCTCGATTCAT-3′	131	60
TCONS_l2_00020565	5′-GTGGCAACTGTGACATTTATC-3′	5'-ATGTCGGACATAACTAATCCC-3′	103	60
TCONS_00014512	5′-GGGTCTCAGCACATATTCC-3′	5′-CCCGCAGTTACCTACATTT-3'	106	60
ESM1	5′-AAAGGCTGCTGATGTAGT-3′	5′-TCTCTGAGGTGGCATACG-3′	108	60
GAPDH	5′-TGTTGCCATCAATGACCCCTT-3′	5′-CTCCACGACGTACTCAGCG-3′	201	60

### Statistical analyses

All data were expressed as mean ± standard deviation. Statistical analysis was performed using Student's *t*-test to compare two variables of microarray data. For example, the statistical significance of a microarray result was analyzed by fold change, and a difference with *P*<0.01 was considered statistically significant. The false discovery rate was also calculated to correct the *P* value. The threshold value we used to screen differentially expressed lncRNAs and mRNAs was a fold change ≥2.0 (*P*<0.01). Furthermore, a differential expression of each lncRNA between hepatoblastoma and the paired distant noncancerous tissues was analyzed using Student's *t*-test with SPSS (Version 16.0 SPSS, Chicago, IL, USA). *P*<0.05 was considered significant.

## Results

### Differentially expressed lncRNAs in hepatoblastoma tissues

**Figure 1:**
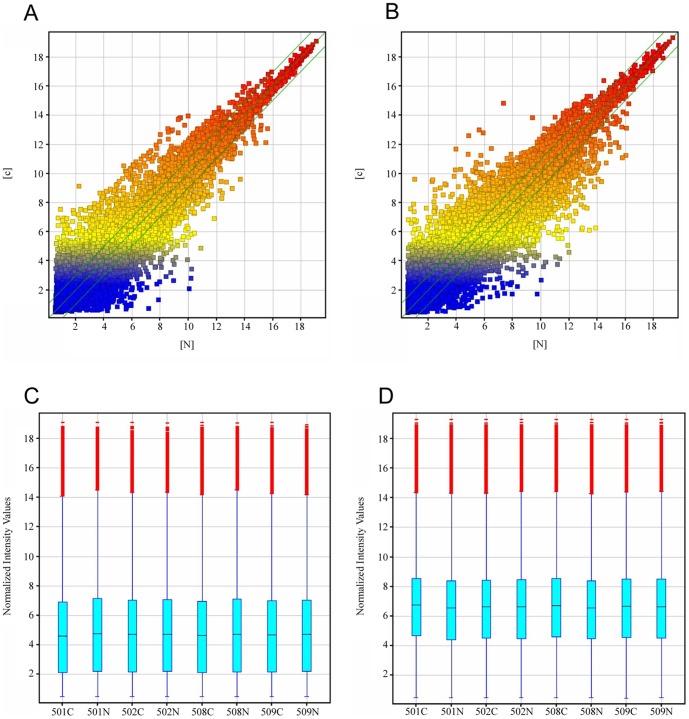
Differentially expressed lncRNAs and mRNAs in 4 hepatoblastoma vs. paired distant noncancerous tissues. The box plot is a convenient way to quickly visualize the distributions of a dataset of lncRNA (A) and mRNA (B) profiles. After normalization, the distributions of log_2_ ratios among nine samples were nearly the same. The scatter plot is a visualization method used to assess the lncRNA (C) and mRNA (D) expression variations between hepatoblastoma and the paired distant noncancerous tissues. The values of the X- and Y-axes in the scatter plot are the averaged normalized signal values of the group (log_2_ scaled). The green lines represent fold change lines (the default fold change given is 2.0).

To profile differentially expressed lncRNAs in hepatoblastoma, we performed a genome-wide analysis of lncRNA and mRNA expression in hepatoblastoma and matched normal tissues (Figure 1). Using the authoritative data sources, we first assessed the lncRNA expression profiles in hepatoblastoma vs. the paired distant noncancerous tissues. We found that 2736 lncRNAs were differentially expressed (fold change ≥2.0, *P*<0.01) between hepatoblastoma and the paired distant noncancerous tissues. Among them, 1757 lncRNAs were upregulated (more than two-fold in hepatoblastoma vs. the normal tissues) and 979 lncRNAs were downregulated (more than two-fold; *P*<0.01; Table S1).

### Differentially expressed mRNAs in hepatoblastoma tissues

Using the differentially expressed lncRNAs data (above), we predicted the expressions of potential target mRNAs, since lncRNAs participate in transcriptional, epigenetic, or post-transcriptional regulation of gene expression. To predict their target genes, we constructed an expression profile of the mRNAs using these differentially expressed lncRNAs. First, we performed a bioinformatics analysis to predict potential lncRNA targets in the database, using target prediction programs. We then integrated the predicted potential lncRNA targets with the differently expressed mRNAs in the profile (fold change ≥2.0, *P*<0.01).

In this way we found that 3060 mRNAs were targeted by these lncRNAs and could be differentially expressed between hepatoblastoma and the paired distant noncancerous tissues. Among them, 2122 mRNAs were upregulated in hepatoblastoma, while 938 mRNAs were downregulated, both more than two-fold (*P*<0.01; Table S2). We found 420 matched lncRNA-mRNA pairs for 120 differentially expressed lncRNAs and 167 differentially expressed mRNAs (Table S3).

### Construction of the co-expression network using GO and pathway analyses

**Figure 2:**
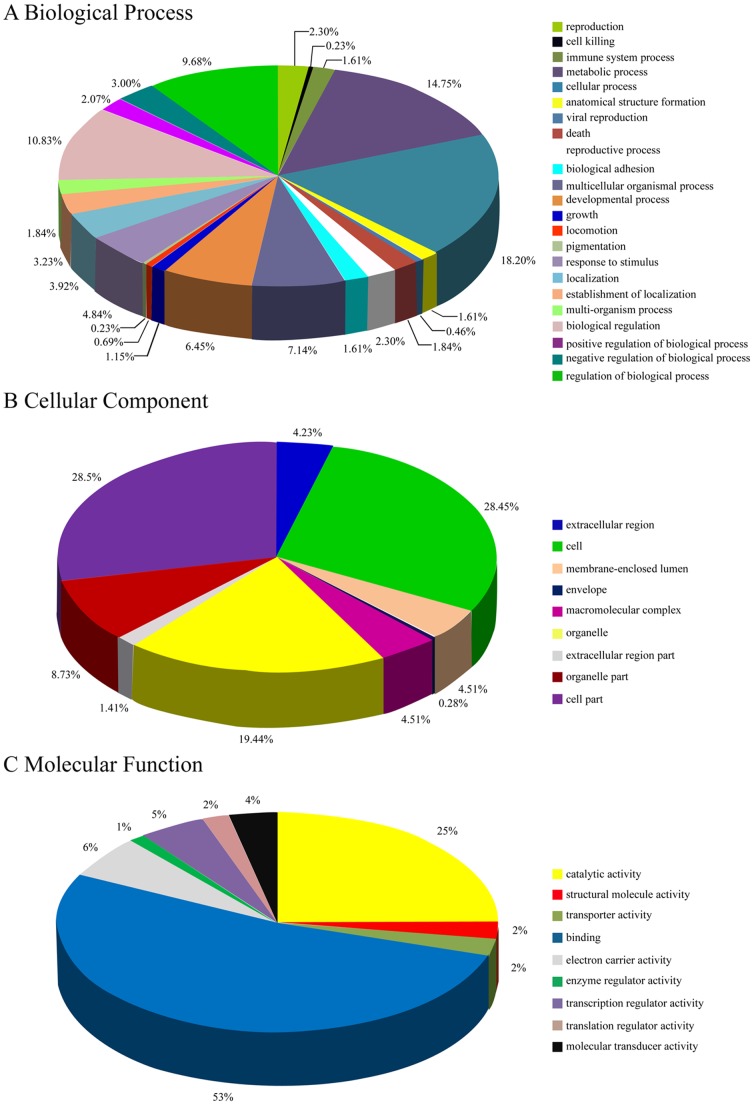
Gene ontology (GO) enrichment analysis of lncRNA-targets. (A) GO analysis of lncRNA-target genes according to biological process. (B) GO analysis of lncRNA-target genes according to cell component. (C) GO analysis of lncRNA-target genes according to molecular function.

Through GO analysis we found that these under-regulated and upregulated transcripts of lncRNAs were associated with cellular process (ontology: biological process), cell (ontology: cellular component), and binding (ontology: molecular function) (Figure 2 and Table S4). Pathway analysis determined that these lncRNAs may target 22 gene pathways that corresponded to transcripts, i.e., “Drug metabolism - other enzymes” composed of six targeted genes (with the recommended *P*-value = 0.05) and “metabolic pathways” (Figure 2 and Table S5). The latter has been reported previously to be involved in hepatoblastoma development [18].

**Figure 3:**
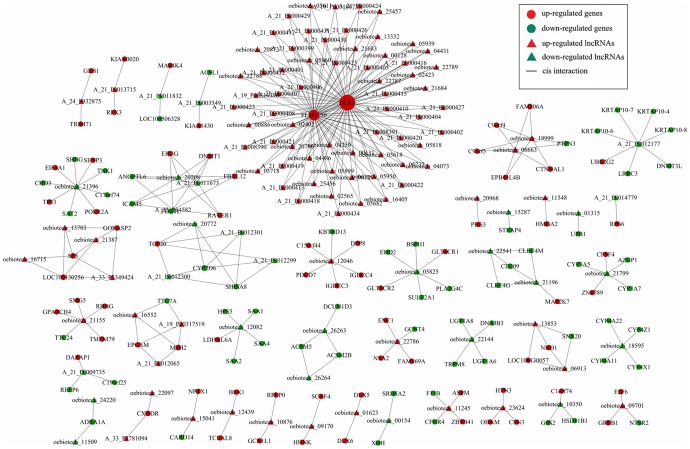
Prediction of lncRNA-mRNA association network. The co-expression network was composed of 252 network nodes and 420 connections between 120 lncRNAs and 132 coding genes. Within this co-expression network, 369 pairs presented as positive, and 51 pairs presented as negative. This co-expression network indicated that one lncRNA could target 11 coding genes at most and that one coding gene could correlate with 128 lncRNAs at most.

Subsequently, we constructed a co-expression network of these coding-noncoding genes that included the differentially expressed lncRNAs and targeted coding genes. We used Pearson's correlation coefficients, equal to or greater than 0.95, to identify the lncRNAs and coding genes and drew the network using Cytoscape. Our data showed that the co-expression network was composed of 252 network nodes and 420 connections between 120 lncRNAs and 132 coding genes. Within this co-expression network, 369 pairs presented as positive, and 51 pairs presented as negative. This co-expression network indicated that one lncRNA could target, at most, 11 coding genes and that one coding gene could correlate with at most 128 lncRNAs (Figure 3).

### qRT-PCR validation of the co-expression network genes

We randomly selected six upregulated lncRNAs (TCONS-00090092-MEG3, TCONS-l2-00000179, TCONS-l2-00014091, TCONS-l2-00004424, TCONS-l2-00021262, and TCONS-00014978) and under-regulated lncRNAs (TCONS-l2-00018070, TCONS-l2-00018071, TCONS-l2-00006843, TCONS-l2-00030560, TCONS-l2-00020565, and TCONS-00024647) as well as a paired lncRNA and mRNA (TCONS-00014512 vs. ESM1) for verification in these four hepatoblastoma patients.

**Figure 4:**
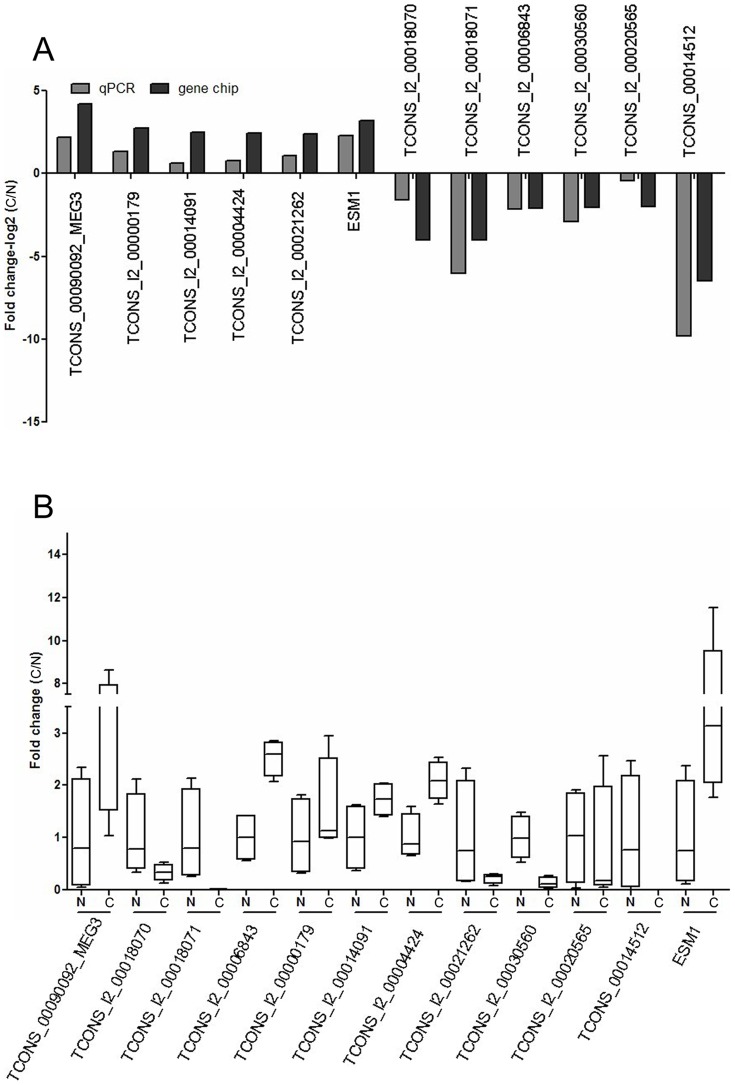
qRT-PCR validation of some differentially expressed lncRNAs and ESM1 mRNA in these 4 hepatoblasoma tissue samples. The data showed that expressions of lncRNAs TCONS-00090092-MEG3, TCONS-l2-00000179, TCONS-l2-00014091, TCONS-l2-00004424, TCONS-l2-00021262, and TCONS-00014978 and ESM1 mRNA were upregulated and that TCONS-l2-00018070, TCONS-l2-00018071, TCONS-l2-00006843, TCONS-l2-00030560, TCONS-l2-00020565, TCONS-00024647, and TCONS-00014512 were downregulated in hepatoblastoma tissues relative to the paired distant noncancerous tissues, consistent with the microarray data.

The results showed that expression of TCONS-00090092-MEG3, TCONS-l2-00000179, TCONS-l2-00014091, TCONS-l2-00004424, TCONS-l2-00021262, TCONS-00014978 and ESM1 were over-regulated, and TCONS-l2-00018070, TCONS-l2-00018071, TCONS-l2-00006843, TCONS-l2-00030560, TCONS-l2-00020565, TCONS-00024647, and TCONS-00014512 were downregulated in all four hepatoblastoma tissue samples relative to the paired distant noncancerous tissues (*P*<0.05; Figure 4, Figure S1).

## Discussion

For the past two decades the molecular mechanisms responsible for hepatoblastoma development have been extensively studied, yet the pathogenesis of this disease is still vague and most of the altered gene expression and regulation involved remain to be delineated. miRNA is another class of noncoding small RNA and plays an important role in tissues and cells, such as embryo development and immunologic response. Altered expression of miRNAs contributed to human disease and carcinogenesis [19]–[21]. Nevertheless, although lncRNA also is non-protein coding RNA, they are longer than 200 nucleotides. However, to date, the majority of lncRNAs are likely to be functional, but only a relatively small proportion has been demonstrated to be biologically relevant, such as regulation of basal transcription machinery functions in cells, RNA splicing and translation, like miRNA, and gene epigenetic regulation [22],[23]. As whole, the functions of lncRNA in cells are less clear compared to those of miRNAs. Thus, we conducted the current study to better understand the role of lncRNA in hepatoblasoma development.

Recently, increasing evidence has shown that lncRNAs are important factors controlling gene expression [24], *cis* (i.e., on neighboring genes) or *trans* (distant genes). The specifics however are not easily predicted based on lncRNA sequence [25], [26]. Of all the functions of which they have been implicated, the most important is their involvement in tumorigenesis, such as in live cancer [27]. Previous studies have shown that regulation of the expression of the p53 gene is through the lncRNA maternally expressed 3 (MEG3) [28], [29], and the latter is commonly altered in hepatoblastoma [30], [31]. Thus, the aberrant expression of at least one lncRNA has been linked to hepatoblastoma development.

To date, there has been no report of differentially expressed lncRNAs in hepatoblastoma tissues. Our data is the first to show a total of 2736 differentially expressed lncRNAs in hepatoblastoma tissues, with fold changes of 2 or more. We then found 420 matched lncRNA-mRNA pairs for 120 differentially expressed lncRNAs and 167 differentially expressed mRNA. GO and pathway analyses predicted that downregulated and upregulated transcripts of lncRNAs were associated with cellular process (ontology: biological process), cell (ontology: cellular component), and binding (ontology: molecular function), which associated with 22 gene pathways that corresponded to transcripts, e.g., “Drug metabolism - other enzymes” composed of six targeted genes (at recommended *P*-value = 0.05) and “metabolic pathways”. The latter has been reported before to be involved in hepatoblastoma development [18].

The co-expression network analysis showed a total of 252 network nodes and 420 connections between 120 lncRNAs and 132 coding genes. Within this co-expression network, 369 pairs presented as positive, and 51 pairs presented as negative. In addition, we verified the presence of some of these differentially expressed lncRNAs and ESM1 mRNA in hepatoblastoma tissues. The data from the current study shows that expression of these altered lncRNAs could contribute to hepatoblastoma development. Further study of these lncRNAs could provide useful insight into hepatoblastoma biology.

Indeed, a downregulated lncRNA (i.e., TCONS-00014512) in hepatoblastoma tissues was found to be located near ESM1. ESM1 functions to promote cell survival, cell cycle progression, tumor cell migration and invasion, and tumor angiogenesis and may serve as a tumor biomarker to predict survival of cancer patients, making ESM1 a potential therapeutic target [32], [33], [34], [35]. A natural antisense association between downregulated lncRNA TCONS-00014512 and ESM1 may help us learn more about how lncRNAs regulate gene expression in hepatoblastoma. Upregulated lncRNA TCONS-00090092-MEG3 is important in the regulation of proper cell growth and embryo development and therefore may be a putative tumor suppressor gene, because one of its functions is to activate p53 and inhibit cell proliferation [36], [37], [38]. *Meg3* can also control gene expression at imprinted loci through recruitment of the polycomb repressive complex 2 (PRC2) complex [39].

To understand the functions of lncRNAs further, in the current study we applied pathway analysis to associate these differentially expressed lncRNAs with their target genes and found that 22 pathways corresponded to transcripts; the most enriched network was ‘Drug metabolism - other enzymes’ composed of 6 targeted genes. One of these pathways, the gene category ‘metabolic pathways’, has been reported to be involved in the development of hepatoblastoma [18].

The current study of lncRNAs in hepatoblastoma tissues is a proof-of-principle that lncRNAs have a probable role in hepatoblastoma development and progression. Hepatoblastoma is an uncommon malignant liver neoplasm of children, and its etiology, pathophysiology, and molecular mechanism is largely unknown. Many more studies are needed to fully understand this disease to effectively control it in the future. Our current study on the potential link between lncRNAs and hepatoblastoma presents a novel area for further investigations into the target genes of such lncRNAs, leading to therapeutic strategies for the disease.

## Supporting Information

Figure S1 qRT-PCR validation of some differentially expressed lncRNAs and ESM1 mRNA in hepatoblasoma tissues.
(TIF)

Table S1 Differentially expressed lncRNAs in hepatoblastoma tissues. (more than two-fold; *P*<0.01).
(XLSX)

Table S2 Differentially expressed mRNAs in hepatoblastoma tissues. (more than two-fold; *P*<0.01).
(XLSX)

Table S3 The matched lncRNA-mRNA pairs for differentially expressed lncRNAs and mRNAs.
(XLSX)

Table S4 Functional classification of the target genes by GO analysis.
(XLSX)

Table S5 Pathway Enrichment analysis.
(XLSX)
