# Supplementary figures and images for: Genome-Wide Analysis of Long Noncoding RNA (lncRNA) Expression in Hepatoblastoma Tissues
Source: PLoS One. 2014 Jan 17;9(1):e85599. doi: 10.1371/journal.pone.0085599 (PMC3894996; doi:10.1371/journal.pone.0085599)

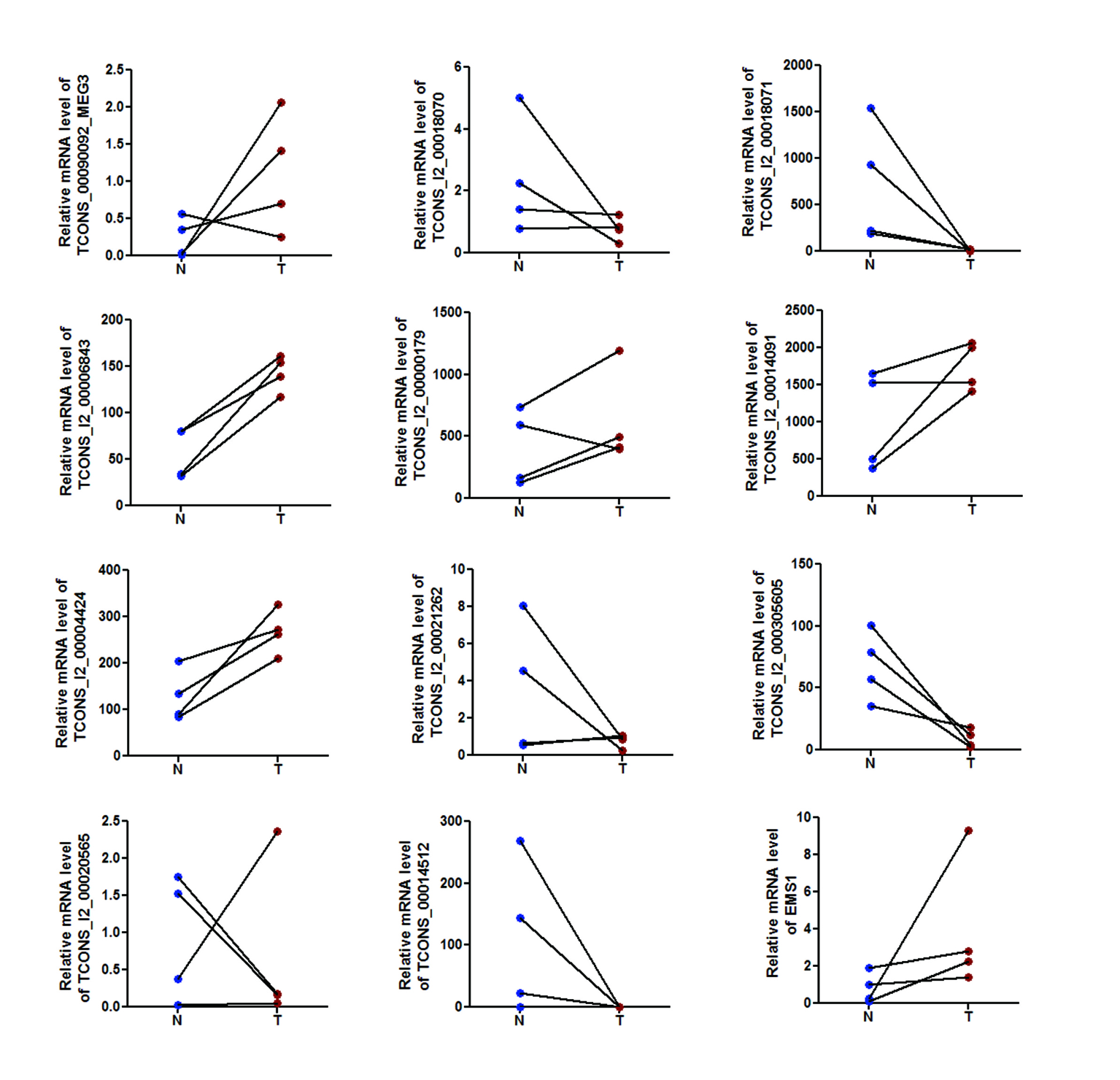

Supplement: Figure S1 — qRT-PCR validation of some differentially expressed lncRNAs and ESM1 mRNA in hepatoblasoma tissues. (TIF) [file pone.0085599.s001.tif]
